# Towards Non-Invasive Diagnosis of Skin Cancer: Sensing Depth Investigation of Open-Ended Coaxial Probes †

**DOI:** 10.3390/s21041319

**Published:** 2021-02-12

**Authors:** Cemanur Aydinalp, Sulayman Joof, Tuba Yilmaz

**Affiliations:** Department of Electronics and Communication Engineering, Istanbul Technical University, Istanbul 34469, Turkey; aydinalp16@itu.edu.tr (C.A.); joof@itu.edu.tr (S.J.)

**Keywords:** microwave dielectric spectroscopy, open-ended coaxial probe, sensing depth, skin cancer detection

## Abstract

**Simple Summary:**

This work investigates the effect of skin tissue heterogeneity on the sensing depth of the open-ended coaxial probe to exploit the potential use of the probe for skin cancer detection and to establish a simple measurement protocol for skin depth characterization. Skin depth was calculated through simulations and measurements. Heterogeneity was obtained using double-layered materials composed of gel-like skin mimicking material and liquid olive oil, triton X-100. It was concluded that the sensing depth was not dependent on the frequency between 0.5 to 6 GHz, was affected by the material located at the aperture of the probe, and lastly the dielectric property contrast between layers. Namely the degree of heterogeneity affects the probe sensing depth.

**Abstract:**

Dielectric properties of biological tissues are traditionally measured with open-ended coaxial probes. Despite being commercially available for laboratory use, the technique suffers from high measurement error. This prevents the practical applications of the open-ended coaxial probes. One such application is the utilization of the technique for skin cancer detection. To enable a diagnostic tool, there is a need to address the error sources. Among others, tissue heterogeneity is a major contributor to measurement error. The effect of tissue heterogeneity on measurement accuracy can be decreased by quantifying the probe sensing depth. To this end, this work (1) investigates the sensing depth of the 2.2 mm-diameter open-ended coaxial probe for skin mimicking material and (2) offers a simple experimental setup and protocol for sensing depth characterization of open-ended coaxial probes. The sensing depth characterized through simulation and experiments using two double-layered configurations composed to mimic the skin tissue heterogeneity. Three thresholds in percent increase of dielectric property measurements were chosen to determine the sensing depth. Based on the experiment results, it was concluded that the sensing depth was effected by the dielectric property contrast between the layers. That is, high contrast results in rapid change whereas low contrast results in a slower change in measured dielectric properties. It was also concluded that the sensing depth was independent of frequency between 0.5 to 6 GHz and was mostly determined by the material located immediately at the aperture of the probe.

## 1. Introduction

Over the past half-century, researchers showed a great interest in quantifying the microwave dielectric properties of biological tissues. This effort paved the way for the development of microwave diagnostic and therapeutic technologies [1,2,3,4,5]. Dielectric properties of biological tissues are pivotal design parameters for the development of such devices [6,7,8,9]. Among other dielectric property measurement techniques, open-ended coaxial probe technique has been a widely preferred data collection method due to several advantages including non-destructive measurement features, broadband measurement capabilities, and flexible sample requirements [10,11,12,13]. Therefore, the technique is widely utilized to quantify the in vivo and ex vivo dielectric properties of different biological materials. For example, in [7], open-ended coaxial probe technique was utilized to measure dielectric properties of excised animal tissue, human autopsy materials, and in vivo dielectric properties of human skin, tongue tissue between 10 Hz and 20 GHz. This study reported a comprehensive data reservoir of biological tissue dielectric properties. In another study, ex vivo dielectric properties of normal, benign, and malignant breast tissues were analyzed between 0.5 to 20 GHz [12,14]. More recently, practical applications of open-ended coaxial probe technique was envisioned one such application is the utilization of the technique as a biopsy device [15,16]. Even though open-ended coaxial probe technique has a number of advantages, the technique suffers from high error rates that prevent the launch of the technique to clinical practice as a medical device. High error rates can stem from equipment or tissue related variability. Equipment related errors can result from the equipment of choice, measurement uncertainties, and calibration [17]. Tissue related errors are caused by temperature discrepancies, probe-sample contact, probe contact pressure, in vivo versus ex vivo experiments, sample handling procedure, tissue sample properties, and tissue heterogeneity. Reported in vivo tissue and phantom dielectric property comparisons show that the sample heterogeneity has a large effect on tissue-related errors [16]. To mitigate the tissue-related errors, there is a need to define the relationship between the sample and probe sensing depth.

Several studies were presented in the literature attempting to characterize the sensing depth of the open-ended coaxial probes. In [18], two probes with 18 mm and 21 mm aperture diameters were used to analyze the probe aperture dependent effective penetration depth for heterogeneous tissue samples. In another study, the sensing volume of two open-ended coaxial probes with 2.2 mm and 3.58 mm aperture diameters were examined for measurement of breast tissue dielectric properties [19]. In [20], sensing the volume of open-ended coaxial probe with 2.2 mm-diameter was investigated by using double-layered configuration composed of liquid and Teflon to represent the heterogeneous tissue composition. In [21], sensing depth of open-ended coaxial probe with 2.2 mm aperture diameter was investigated by performing experiments with six double-layered configurations based on analysis of five different sensing depth definitions. These studies used different terminologies such as effective penetration depth, sensing volume, histology region, sensing depth to describe the relationship between sample heterogeneity and measured dielectric properties with open-ended coaxial probes [18,19,20,21,22]. In [19], sensing volume was explained as the smallest distance between the probe and boundary (beaker, stand and air) for which the real ($\epsilon^{\prime }$) and imaginary ($\epsilon^{\prime \prime }$) parts of the complex permittivity errors remain below 10%. In [20], sensing volume was described as the thickness of intervening liquid when the measured dielectric properties reached 50 and 90%. For a double-layered configuration, histology region or depth was described as the distance where the contribution of the second layer’s dielectric properties to the measured dielectric properties of the first layer becomes undetectable [21]. Reported studies in [18,19,20,21] indicate that the definition of sensing depth varies based on measurement sample, probe aperture size and the chosen measured dielectric property threshold (variation of dielectric property as a percentage). While these analyses are important for understanding the relationship between sensing depth and dielectric properties of heterogeneous tissues, these studies must be expanded by including specific tissue types to realize the practical applications of open-ended coaxial probes. Further, there is a need to establish a robust and replicable measurement protocol to quantify the sensing depth. To this end, the goal of this work is to investigate the sensing depth of a commercially available open-ended coaxial probe with 2.2 mm aperture diameter for skin cancer detection in order to,

- define the sensing depth of the probe for skin tissue by utilizing skin mimicking materials,
- establish a simple measurement protocol to quantify the sensing depth of open-ended coaxial probes.

This work attempts to minimize the potential measurement errors to enable an open-ended coaxial probe-based skin cancer detection device. To do so, two double-layered sample configurations were prepared to represent the heterogeneous tissues. Considering that many different definitions were used to describe this measurement concept, we used the term “sensing depth” to define the measurement sensitivity; that is, the intervening liquid thickness where the measured dielectric properties changes by 5% of the pure liquids’ dielectric properties. In this study, we also chose three thresholds (reflecting the percent change) for sensing depth measurement. The sensing depth of open-ended coaxial probe with 2.2 mm aperture diameter (Keysight Technologies, Santa Rose, CA, USA) was investigated utilizing the heterogeneous double-layered samples. The remainder of this paper is organized as follows: Section 2, materials and methods, simple and easily replicable measurement setup and protocol are explained in detail. In Section 2 simulation configurations are described. Section 3 shows results obtained from both experiments and simulations. In Section 4, obtained results are discussed in detail.

## 2. Materials and Methods

In the following section, the details of dielectric property measurements and simulations are explained. Experiment setup, prepared skin mimicking phantom material, double-layered sample arrangements and measurement protocol along with simulation configurations are given.

### 2.1. Experiment Setup

**Figure 1:**
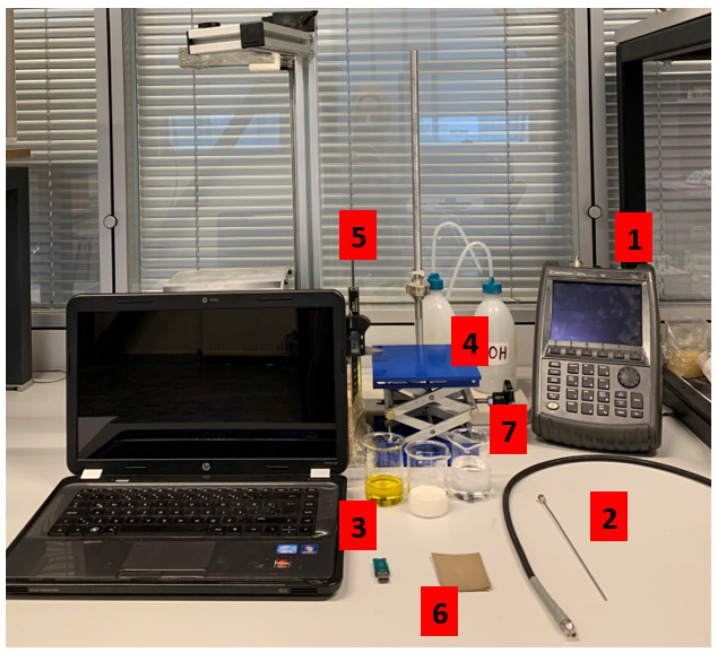
Experimental setup: (1) Agilent FieldFox N9923A 6 GHz RF Vector Network Analyzer (VNA), (2) Agilent N1501A Dielectric Slim Form Probe, (3) Agilent 85070E software, (4) an adjustable stand, (5) a digital caliper (6) conductive textile and (7) pure material samples (olive oil, skin phantom and triton X-100).

Dielectric property measurement setup consisted an Agilent FieldFox N9923A 6 GHz RF Vector Network Analyzer (VNA) (Santa Clara, CA, USA), an Agilent dielectric slim form probe with 2.2 mm aperture diameter, Agilent 85070E dielectric property measurement software (Santa Clara, CA, USA), an external computer, an adjustable stand, and a digital caliper. The experiment setup is shown in Figure 1. Open-ended coaxial probe with 2.2 mm aperture is frequently preferred for dielectric property measurements of biological tissues and high permittivity, high loss liquid or gel-like materials; therefore, the probe was selected for this work [11,15].

The (VNA) was used to collect the S parameter response between 0.5 and 6 GHz with 55 MHz increments. This frequency range was chosen based on the limitation of the VNA and also many biological tissue studies were performed in this frequency range. Agilent 85070E software was installed on the external computer for dielectric property calculation from the measured S parameter response of the probe. The calibration process was carried out according to the software instructions: open circuit (the probe tip was left in the open air), short circuit (the probe tip was terminated with a conductive textile), a broadband load (the probe tip was immersed in distilled water with known temperature). In order to eliminate temperature drift errors caused by thermal expansion characteristics, the experiment setup was turned on 4 h prior to measurements. Mitutoyo absolute digimatic caliper 0–150 mm with 0.01 mm digital step size was used to measure the probe tip distance from layers.

### 2.2. Sample Configuration

**Figure 2:**
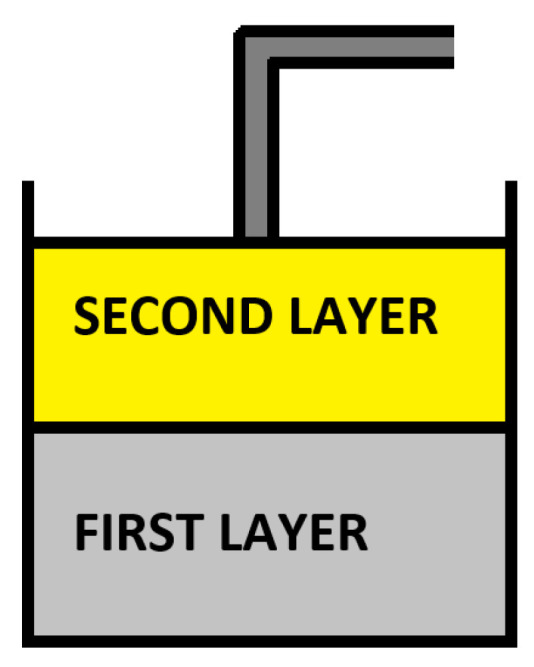
Diagram of the double-layered configuration consisting of skin mimicking phantom as the first layer and olive oil or triton X-100 as the second layer.

Sensing depth of 2.2 mm-diameter open-ended coaxial probe was analyzed by preparing two different double-layered sample configurations; that is, skin mimicking phantom-olive oil and skin mimicking phantom-triton X-100 as shown in Figure 2. Although the prepared samples were heterogeneous, each layer was individually homogeneous. The first layer (skin mimicking phantom) was placed at the bottom of the configuration while the second layer (olive oil or triton X-100) was at the top of the configuration. Note that the composed phantoms are chemical gels and once jellified it can not be liquified again. Therefore, we do not expect an interaction, chemical or otherwise, between the liquids and the phantom. Throughout the experiments, the samples were inspected by eye to ensure that the second layer was visibly clear.

**Figure 3:**
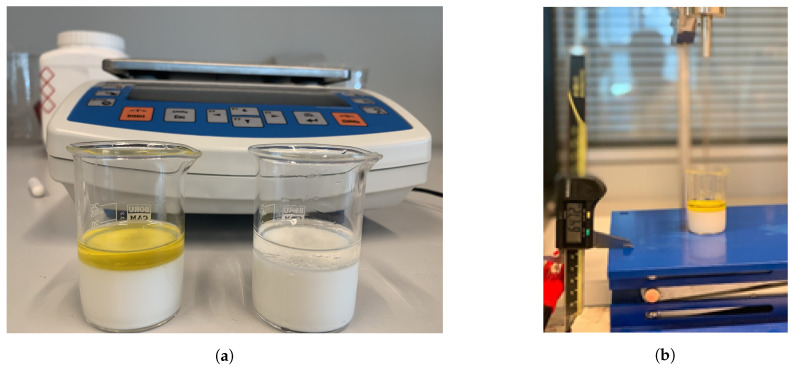
Double-layered configurations and part of the experiment setup for the measurement of layer thickness and step size: (**a**) skin phantom-olive oil (left) and skin phantom-triton X-100 (right), (**b**) adjustable stand and digital caliper for thickness measurements.

The details of the skin mimicking phantom recipe and preparation along with a comparison to skin tissue dielectric properties were given in [23]. The homogeneity of the phantom was evaluated by measuring at seven different depths from the surface to the bottom of a cylindrical shaped phantom. The phantom dimensions were 65 by 65 mm in diameter and height, respectively. At each depth, five different positions were measured. In total, the standard deviation from the mean of the measurement results taken from 35 different points on the skin-mimicking phantom was found to be 0.428 at 0.5 GHz, which indicated that the phantom was homogeneous enough to be utilized as the first layer. For the double-layered configurations; first, 15 mL of liquid phantom was solidified in a beaker then the desired liquid was added as the second layer. Prepared double-layered configuration samples are shown in Figure 3. Measured dielectric properties of each layer at 0.5 and 4.02 GHz frequency points are listed in Table 1.

### 2.3. Measurement Protocol for Sensing Depth Characterization 

**Figure 4:**
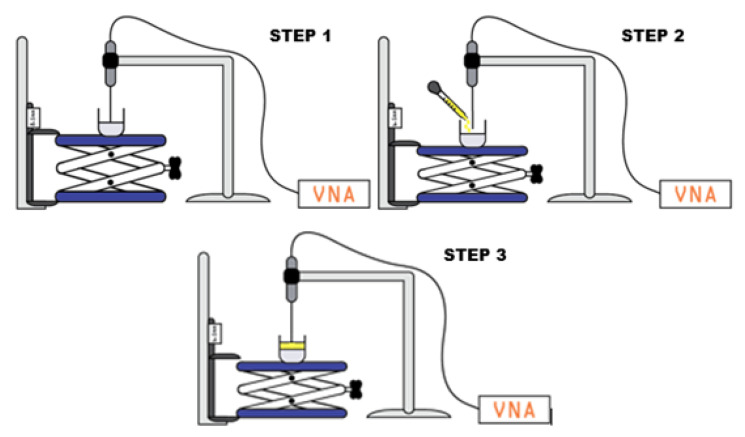
The process of the dielectric property measurement for sensing depth analysis.

The experimental procedure utilized in order to investigate the sensing depth of the 2.2 mm open-ended coaxial probe, shown in Figure 4, is explained as follows:

- Figure 4 Step 1:
  – The probe was placed in a fixed position and the same position was maintained throughout the measurement procedure in order to reduce the error due to cable movement.
  – The first layer was placed on the adjustable stand platform and slowly lifted towards the probe tip.
  – When the first layer’s surface reached the probe tip, the caliper reading was recorded as the reference distance value for the second layer.
- Figure 4 Step 2:
  – The adjustable stand was lowered in order to add the second layer.
  – Without changing the position of the sample, the liquid second layer was added using a Pasteur pipette.
  – Similar to step 1, when the top surface of the liquid second layer reached the tip of the probe, the value displayed on the caliper was recorded as the second reference distance value. Note that the difference between the two reference points gives the thickness of the second layer and the measurement step size was determined accordingly. The knowledge of the first reference point eliminates the changes in measured distance due to probe pressure to gel-like the first layer. This simple approach prevents a potential misleading sensing depth measurement.
- Figure 4 step 3:
  – The first dielectric property measurement was taken when the probe tip was touching the top surface of the second layer; that is, the second reference point.
  – Next, the double-layered sample was gradually lifted with the adjustable stand allowing the probe to immerse into the second liquid layer. The dielectric property measurement was collected for each probe position at different depths in the liquid second layer.
  – Experiments were finalized when the probe tip reached the top surface of the first layer; that is, the first reference point.

### 2.4. Simulation Configuration for Sensing Depth Characterization

**Figure 5:**
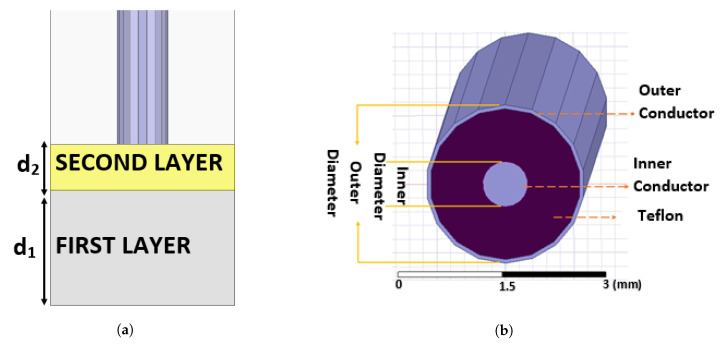
Double-layered simulation configuration and 2.2 mm-diameter probe design: (**a**) first layer represents the skin tissue with d_1_ thickness and the second layer represents the liquid (olive oil or triton X-100) with d_2_ thickness. (**b**) Simulated open-ended coaxial probe with 2.2 mm outer diameter and 0.657 mm inner diameter.

Open-ended coaxial probe and two different double-layered sample configurations were simulated with Ansys High-Frequency Structure Simulator(HFSS), 3D electromagnetic (EM) simulation software as shown in Figure 5. The commercially available Agilent’s 2.2 mm probe datasheet, provides only the outer diameter of the probe. This probe was replicated using the same outer diameter. Teflon ($\epsilon_{r}$ = 2.1) is used as the dielectric between the inner and outer conductor. For a coaxial probe with an outer diameter of 2.2 mm and Teflon as the dielectric material, the diameter of the inner conductor was calculated as 0.657 mm for 50 Ω impedance matching. Two different double-layer configurations were configured by assigning skin tissue for the first layer and olive oil or triton X-100 for the second layer. The simulations were performed in the frequency range of 0.5 to 20 GHz with 0.25 GHz increments by selecting various top layer thicknesses (d${}_{2}$) with three different ranges: 0.01–1 mm with 0.05 mm increments, 1–3 mm with 0.2 mm increments and 3–5 mm with 1 mm increments. The main reason for determining three different ranges was to reduce the simulation time and to be able to work with small increments in the range where the relative permittivity alteration was highest. Note that the sensing depth is determined based on the percent change in relative permittivity. Therefore, it is important to collect probe response in smaller distances when the measured dielectric property change could be significant. To retrieve complex dielectric properties from simulated S parameter responses, an in-house algorithm was used. Validation of the in-house algorithm was performed by comparing the literature, simulation, and experiment results for skin tissue that are listed in Table 2. For the simulation, the Debye parameters $\epsilon_{\infty }$, $\epsilon_{s}$ and τ were selected as 4, 42 and 6.9 ps, respectively [24]. Please note that the dielectric properties used in the simulation were obtained from [24] and literature values used for comparison were obtained from [7]. Therefore, conductivity discrepancy in Table 2 represents the discrepancy between the sources in the literature. Nevertheless, the sensing depth characterization is traditionally performed with relative permittivity values due to higher measurement uncertainty of conductivity. Thus, the difference between simulation and literature results for the conductivity in this work was omitted. Broadband validation of the in-house algorithm with various materials is given in Section 3.1.2.

### 2.5. Sensing Depth Determination

Reported studies in the literature are analyzed in this section to determine the sensing depth measurement procedure that will be adopted in this work. Details of the two previously reported studies are listed in Table 3 [20,21]. Both studies employed the commercially available Agilent slim form dielectric probe with 2.2 mm aperture diameter.

In the literature, special experiment setups were designed and used to collect the dielectric property data for sensing depth analysis of the 2.2 mm-diameter probe with double-layered sample configurations. In [20], the double-layered configuration was comprised of Teflon and water for the first and the second layer, respectively. The sensing depth of the probe was determined based on the percentage of the relative permittivity change. The sensing depth for 50% relative permittivity change was calculated as 0.2 mm; that is, the distance of probe tip from the top of the first layer. Please note that 50% relative permittivity change indicates measured relative permittivity was equal to 50% of the second layer’s (water) relative permittivity. Similarly, the sensing depth for 90% relative permittivity change was measured at 0.5 mm. Likewise, this sensing depth indicates measured relative permittivity reached to 90% of the second layer relative permittivity. In [21], the distance (the range 1.2–3.65 mm) was calculated based on the flattening of the change in measured dielectric properties. Both reported studies collected the measurements by lowering the samples; thus, started collecting dielectric property data from the first layer. In this work, the sample was lifted towards the probe; therefore, data collection starts from the second layer.

In this work, the sensing depth determination techniques reported by the studies given in Table 3 are utilized. In accordance with the literature, 5, 20 and 80% change in measured relative permittivity rather than the change in measured conductivity due to higher measurement uncertainty reported for conductivity [21]. The reported error of the commercial systems is 5% [25]. To analyze the sensing depth findings within the reported error range we use 5% as the first threshold for sensing depth analysis. The effective penetration depth for 2.2 mm aperture probe was defined as 20% [20]. Therefore, in this study, we selected the 20% as the second threshold for sensing depth analysis. Similarly, in the literature, a complimentary point was chosen to analyze the sensing dept one example is the 10 and 90% change in measured dielectric properties. Therefore, 80% change was chosen as the third threshold complementary to 20%. Obtained results are given in detail in Section 3.

## 3. Results

This section presents the sensing depth analysis performed on relative permittivity obtained from the simulations and measurements. Axial electric field magnitude obtained from simulations using different configurations are shown in this section. Furthermore, the validation of in-house algorithm is demonstrated for pure materials (triton X-100, olive oil) and skin tissue. Note that these materials were used for double-layered configurations. Sensing depth analysis of 2.2 mm-diameter probe from simulation results using double-layered configurations were performed and three probe positions were designated based on the previously defined percent thresholds. Various frequency points were also considered for sensing depth analysis. In the second part of this section, the experiment results based on probe positions are given. Additionally, sensing depth obtained from two different double-layered configurations are compared to analyze the role of the relative permittivity of the second layer. Lastly, similar to simulations, three positions were determined to specify the sensing depth for experiment results.

### 3.1. Simulation Results

#### 3.1.1. Electric Field Distribution

**Figure 6:**
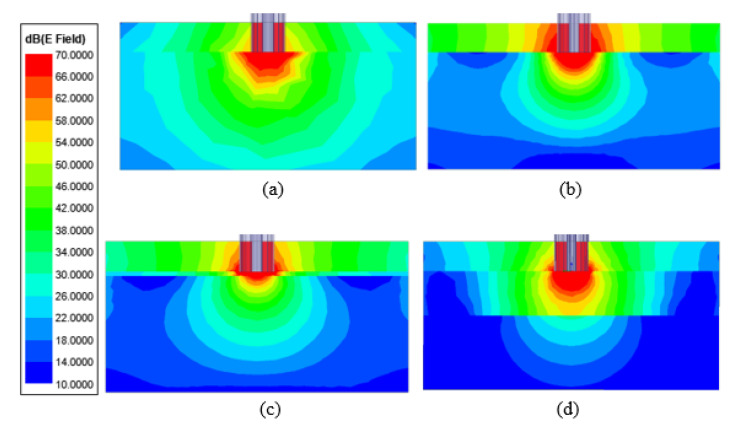
Electric field strength in single layer configurations (**a**) olive oil, (**b**) skin tissue, and double-layered configurations (**c**) olive oil with 0.3 mm thickness, (**d**) olive oil with 3 mm thickness.

The axial magnitude of the electric field at 2 GHz is illustrated in Figure 6a–d when the probe tip is terminated with only skin, only olive oil and two skin–olive oil double-layered configurations. The double-layered configurations include 0.3 and 3 mm olive oil thicknesses. The electric field of these configurations are given directly around the probe tip to precisely demonstrate the field distribution closest to the probe. From the figures, it can be seen that the electric field of the single-layer configurations are different from the double-layered configurations. The electric field was approximately 70 dB around all probe tips, and the electric field magnitude difference between the furthest point and near the probe tip was approximately 60 dB. As seen in Figure 6c, when olive oil thickness was equal to 0.3 mm, the electric field on both layers were approximately 70 dB. When olive oil thickness was selected as 3 mm, the electric field on the skin layer decreased to approximately 30 dB.

#### 3.1.2. In-House Algorithm Validation

Dielectric properties are not directly measurable quantities and in the open-ended coaxial probe technique, these properties are derived from the admittance model [26]. The admittance model relates the ratio of the material’s admittance (Y(ϵ)) and probe’s admittance (Y(0)) to the material dielectric properties via an integral equation. The admittance ratio is calculated from the probe’s reflection coefficient (Γ) which is obtained through the measured S parameter response of the probe when terminated with known materials, so-called calibration, and with the material under test (MUT) as shown in Equation (1).

(1) $$\frac{\left(Y(\epsilon )\right)}{\left(Y(0)\right)}=\frac{\left(1-\Gamma \right)}{\left(1+\Gamma \right)}.$$

Based on the admittance model, the in-house algorithm adopts the Gauss–Newton iterative algorithm with Tikhonov regularization to solve the integral equation. One important aspect of the in-house model is, unlike traditional approaches, the algorithm derives the mathematical model parameters of the MUT dielectric properties instead of solving the admittance model for each frequency. For example, parameters of Debye model or Cole–Cole model. The algorithm solves the admittance model numerically while minimizing the error between the numerical solution (Y’) and measured admittance response (Y) as shown below in Equation (2):

(2) $$\begin{array}{cc} \; & err=abs(Y-Y^{\prime })\\ \; & min\Rightarrow err \end{array}$$

When the solution converges the measured values, the algorithm returns the parameters of the mathematical model representing the complex dielectric properties of the MUT. Note that the input of the algorithm constitutes the simulated or measured S parameter response of the probe with known materials and with MUT.

**Figure 7:**
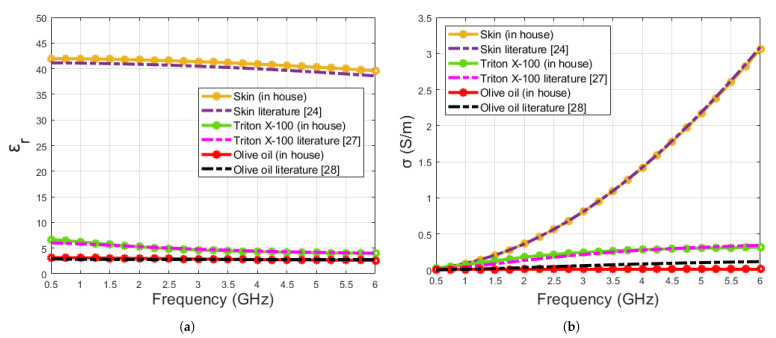
Comparison of pure material (skin, triton X-100 and olive oil) dielectric properties calculated from Debye parameters obtained from the literature and retrieved from the in-house algorithm: (**a**) relative permittivity, (**b**) conductivity.

The proposed in-house algorithm was verified by retrieving the dielectric properties of materials employed in this work namely olive oil, triton X-100 and skin tissue. The following steps were followed for verification, (1) to perform the simulation of the pure materials (skin phantom, triton X-100 and olive oil), the Debye parameters from literature [24,27,28] were given to the simulation, (2) the obtained properties were entered to the simulation program and the S parameter responses of the open-ended coaxial probe were obtained, (3) the simulation results were fed to the in-house algorithm to retrieve a new set of Debye parameters, (4) dielectric properties were calculated by inserting the desired frequency range to the Debye parameters obtained from the in-house algorithm, (5) literature dielectric properties were calculated from the Debye parameters given in [24,27,28] and compared to the retrieved dielectric properties. The comparison of the dielectric properties obtained from the literature [24,27,28] and the in-house algorithm are given in Figure 7. From the figure, it can be seen that the calculated dielectric properties from the Debye parameters retrieved via an in-house algorithm provide good accuracy. Furthermore, the maximum discrepancy between the obtained dielectric properties from the literature data and retrieved dielectric properties from the in-house algorithm at 0.5–6 GHz frequency range are listed in Table 4. The discrepancy was calculated based on absolute values over 0.5–6 GHz frequency range.

#### 3.1.3. Sensing Depth Analysis: Simulation Results

**Figure 8:**
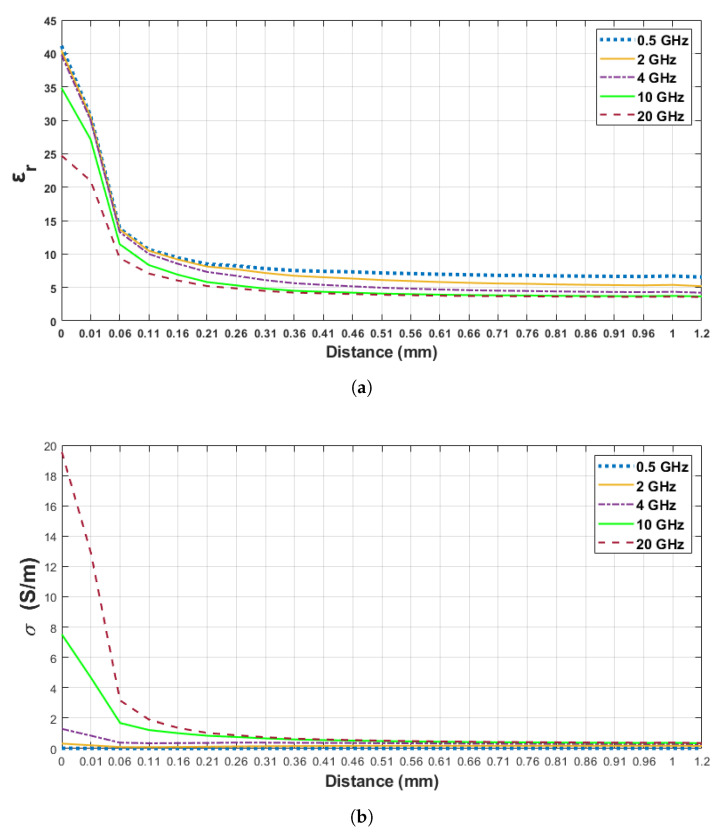
Retrieved dielectric properties of the double-layered skin–triton X-100 configuration: (**a**) relative permittivity, (**b**) conductivity as a function of probe’s distance from first layer at 0.5, 2, 4, 10 and 20 GHz.

In order to analyze the sensing depth of the probe, we selected 5 frequency points between 0.5 and 20 GHz. For analysis purposes, we specified three thresholds for retrieved relative permittivity percent increases as described in Section 2.5. In Figure 8, the change in the dielectric property of the skin–triton X-100 configurations is demonstrated for 0.5, 2, 4, 10 and 20 GHz frequency points against probe distance from the surface of the first layer; that is, the first reference point. Although the sensing depth was analyzed from 0 to 5 mm probe distance, 0 to 1.2 mm probe distance from the first layer is given in Figure 8 for precise graphical representation. In Table 5, increments of 5, 20 and 80% in measured relative permittivity values and corresponding distances at five frequency points are listed. As seen in Table 5, the relative permittivity of triton X-100 increased by 5% between 0.76 and 1.2 mm at five frequency points. As the probe tip approached the first layer, the measured relative permittivity increased by 20% between 0.36 and 0.56 mm probe distances. Between 0.14 and 0.24 mm distances the relative permittivity of the first layer was dominant in double-layered measurement and the retrieved relative permittivity increased by 80% within this region.

**Figure 9:**
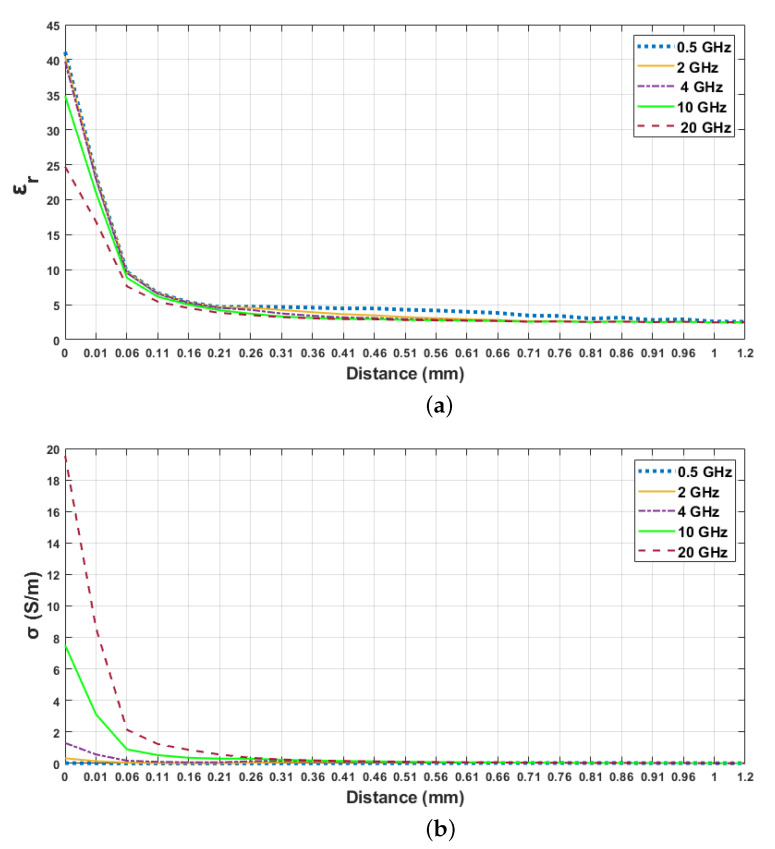
Retrieved dielectric properties of the double-layered skin–olive oil configuration: (**a**) relative permittivity, (**b**) conductivity as a function of probe’s distance from first layer at 0.5, 2, 4, 10 and 20 GHz.

**Figure 10:**
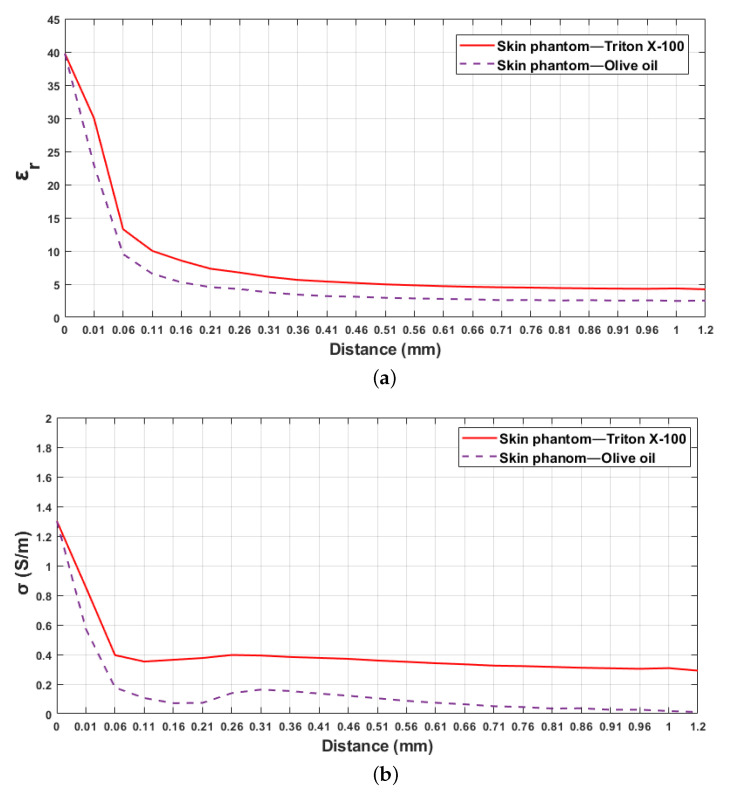
Skin–triton X-100 and skin–olive oil simulated dielectric properties: (**a**) relative permittivity, (**b**) conductivity as a function of probe’s distance from first layer at 4 GHz frequency.

The above-explained simulation design was also performed for skin–olive oil double-layered configuration, where the second layer had lower relative permittivity and conductivity in comparison to skin–triton X-100 configuration. The relative permittivity and conductivity alteration of skin–olive oil configuration are demonstrated in Figure 9a,b, respectively at 0.5, 2, 4, 10 and 20 GHz frequency points. As listed in Table 6, the results obtained are 0.66–1 mm, 0.36–0.86 mm, 0.16–0.36 mm sample thicknesses (distance from the first layer) for 5%, 20%, 80% increase in retrieved relative permittivity, respectively. Figure 10 demonstrates the comparison of double-layered configurations’ dielectric property alterations as a function of probe distance from the first layer (skin) at 4 GHz frequency. In [21], the sensing depth was evaluated in terms of the materials used for double-layered configuration. By comparing the analysis performed in [21] to our results, it can be concluded that the pace of change in retrieved dielectric properties is dependent on the dielectric property contrast between the two layers.

As seen in Figure 10, the change in dielectric properties was gradual for the lower contrast scenario. According to the results given in Table 5 and Table 6 at 2 GHz, the transition from 5 to 80% increase was required more distance for skin–triton X-100 configuration, which verifies the previous finding indicating that sensing depth is dependent on the sample dielectric properties [21]. A more detailed interpretation of sensing depth is given in the following section.

#### 3.1.4. Sensing Depth Analysis: Experiment Results

**Figure 11:**
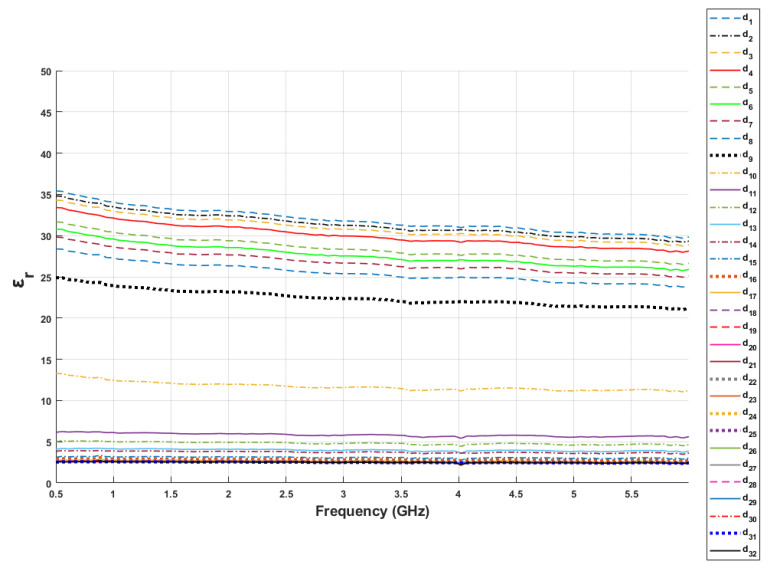
Relative permittivity of skin–olive oil as a function of frequency at various distances between the probe tip and first layer. The distances are expressed as d${}_{1}$ to d${}_{32}$. In d${}_{1}$ position indicates that the skin phantom layer was in full contact with the probe tip.

**Figure 12:**
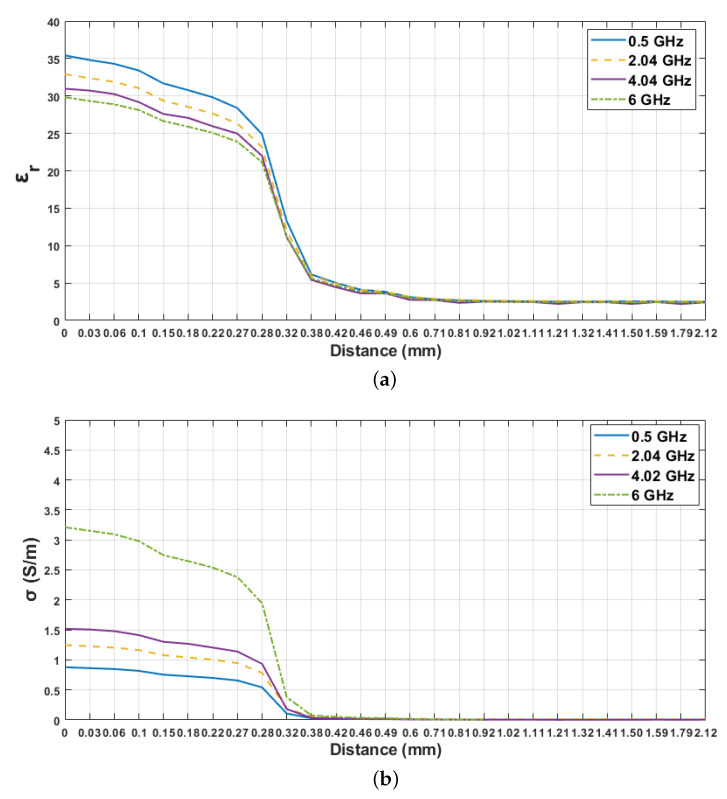
Skin–olive oil dielectric properties as a function of probe distance from the first layer at 0.5, 2.04, 4.02 and 6 GHz frequency points: (**a**) relative permittivity, (**b**) conductivity.

**Figure 13:**
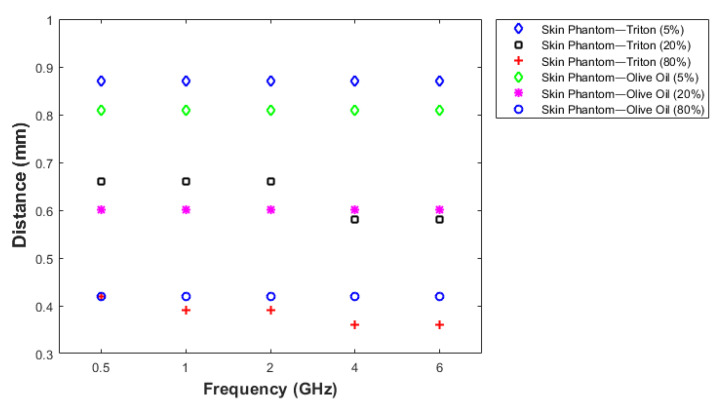
Skin–triton X-100 and skin–olive oil 5, 20 and 80% increase in relative permittivities at 0.5, 1, 2,4 and 6 GHz frequency points.

In order to analyze the sensing depth in detail, the dielectric property measurements were taken by manually adjusting the distance between the probe and the first layer. Measured relative permittivity change with respect to the probe position between 0.5 to 6 GHz is given in Figure 11. In the figure, d${}_{1}$ indicates the value assigned to the point where the probe tip is fully in contact with the first layer (first reference point) and d${}_{32}$ indicates the value assigned to the position where the probe tip is in contact with the second layer (second reference point). The distances from d${}_{1}$ to d${}_{8}$ were 0, 0.03, 0.06, 0.1, 0.15, 0.18, 0.22 and 0.27 mm. At these distances, the dielectric property measurements were similar to single-layer skin phantom configuration, since the thickness of olive oil at the tip of the probe were relatively small. Measured dielectric properties of double-layered configuration varied drastically at d${}_{9}$ (0.28 mm) and d${}_{10}$ (0.32 mm) distances. Even though there is a drastic change between these two points, the measured relative permittivities were still affected by the contribution of the first layer. The measured relative permittivities between 0.38 mm (d${}_{11}$) and 0.49 mm (d${}_{14}$) probe distance varied from 6.14 and 3.84. As seen in Figure 11, smaller dielectric property values are obtained from d${}_{15}$ to d${}_{32}$. In Figure 12, dielectric property changes of these distances for the skin phantom-olive oil configuration are shown in detail by plotting relative permittivity and conductivity versus the distance between the probe tip and the first layer at four different frequency values. Although the thickness of the second layer was 6 mm, the graphs were plotted until 1.32 mm thickness to clearly show the distance axes. Additionally, the sensing depth with three percentage values was analyzed at different frequency points as shown in Figure 13. The sensing depths for previously designated percent change thresholds are given in Table 7 at 0.5, 2.04, 4.02 and 6 GHz frequency points. Note that all percent increases were calculated with respect to dielectric properties of the second layer both in measurements and simulations.

**Figure 14:**
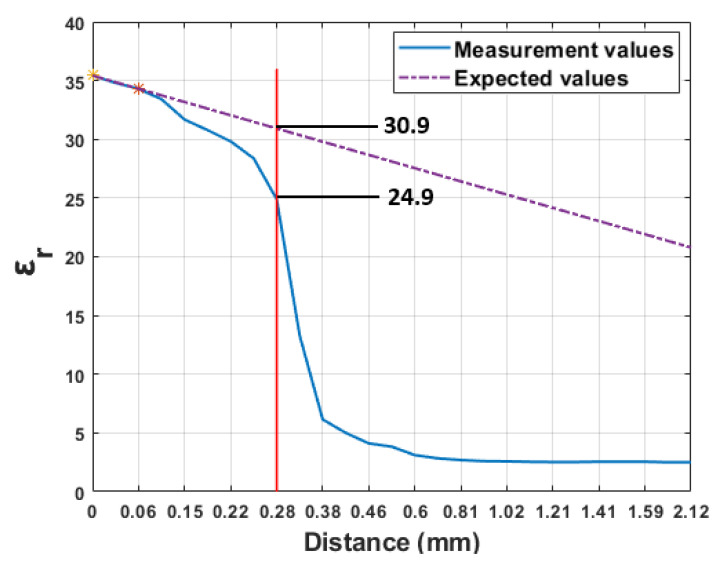
The analysis of relative permittivity changes according to 20% difference between expected and obtained values at 2.04 GHz frequency point for skin–olive oil configuration.

Figure 14 represents the dielectric property of skin–olive oil configuration as a function of probe tip distance from the surface of the first layer (first reference point) at 2 GHz. If the change in the dielectric property was linear, we would have obtained a decreasing linear line, which is shown in Figure 14, obtained by using two points from the measured results. Based on our measurement results, when the distance between the probe tip and the first layer was 0.28 mm, the measured dielectric property change was 20% below the expected value according to the linear line. A similar percentage difference was, also, recorded between the measured and expected value in [18]. The difference between the previous study and this work is at 0.28 mm reported measured dielectric properties were close to the second layer (water $\epsilon_{r}$ = 78.8) in [18] and it is close to the first layer (skin mimicking material $\epsilon_{r}$ = 37.4) in this work. From the obtained results we can state that the medium with a high dielectric property in the close proximity of the probe tip has more impact on the measurements.

## 4. Discussion

Several studies on sensing depth analysis of the coaxial probe have been presented in the literature to overcome tissue and equipment related errors. In this work, application-specific investigation of sensing depth is presented to minimize the dielectric property characterization error for diagnostic purposes. To this end, we characterized a homogeneous skin mimicking phantom for the detection of skin anomalies. A commercially available 2.2 mm aperture diameter probe was used. Double-layered material was prepared by pouring liquid triton X-100 ($\epsilon_{r}$ = 5.86) and olive oil ($\epsilon_{r}$ = 2.56) layers on top of the skin mimicking phantom ($\epsilon_{r}$ = 38.26). Skin–olive oil configuration and the skin–triton X-100 configuration represents high and low dielectric contrast between the layers, respectively. Dielectric properties were collected by gradually lifting the sample towards the tip of the probe. 5 to 80% change in a dielectric property of the triton X-100 layer occurred when the distance between the probe tip and skin phantom top surface was within 1.2–0.17 mm range. A similar percentage change in the dielectric property of the olive oil occurred within 0.81–0.26 mm range for skin–olive oil configuration. From our results, we concluded that the rate of change in measured relative permittivity is approximately 50% faster when there is a high dielectric contrast between the layers. Additionally, the impact of the contrast between two layers can be characterized as: (1) if a large contrast exists between two layers, rapid change in dielectric property measurement is observed, and (2) gradual change in dielectric property is observed if there is a small contrast between two layers.

External factors such as dust, humidity or genetic pre-disposition can cause the formation of a layer above the skin. We investigated the effects of such a layer on the measured dielectric properties of skin tissue by considering the percentage change in measured relative permittivity of a double-layered configuration. This was represented by skin mimicking material and triton X-100 or olive oil in this study. Even though the layer created above the skin by explained factors can be very thin, our study suggests that it will highly affect the dielectric property measurement.

In practice, it is recommended to choose a minimum sample thickness of 5 mm when using the open-ended coaxial probe [25]. Based on our results, we concluded that the surface of the sample under test has more influence on the dielectric property measurements than the size of the sample. For instance, when the thickness of the intervening liquid was 0.28 mm in [18], the measured relative permittivity was 60.6 for Teflon–water double-layered configuration. We obtained a relative permittivity of 24.9 for skin–olive oil configuration at the same distance. The study of sensing depth in [19] was based on 10% error threshold for a dielectric property measurement of ethanol, methanol and water. 10% change in ($\epsilon^{\prime }$) and ($\epsilon^{\prime \prime }$) was observed within 0.75–1.5mm. In [21], the histology region was reported as 1.20–3.65 mm thickness. In this work, 5% increase in the dielectric property of liquid layers (triton X-100 $\epsilon_{r}$ = 5.86 and olive oil $\epsilon_{r}$ = 2.56) was observed within 0.81–0.87 mm the thickness. This work reports a minimum required thickness of 0.81 mm. These results confirm that the material located immediately at the tip of the probe significantly affects the measured dielectric properties.

In [20], when the thickness of the intervening liquid was 0.2 mm, the measured dielectric property reached 50% of liquid’s dielectric property for Teflon–water double-layered configuration. In this work, at 0.22 mm thickness, the measured relative permittivity was 30.7 which is 10 times higher than the dielectric property of intervening liquid. The discrepancy between our results and the previous work can be attributed to the dielectric properties of the materials chosen for each study. In [20], the dielectric property of the intervening liquid was higher than the value of the solid layer; however, in this work, a reverse configuration was employed. Due to the difference in configuration, the dielectric properties of the intervening liquid in [20] was dominant; in contrast, the dielectric property of a solid layer was dominant in our measurement results.

## 5. Conclusions

Knowledge of biological tissue dielectric properties is of paramount importance in the design of microwave medical devices. Therefore, there is a need to accurately characterize the dielectric properties of biological tissues. Tissue heterogeneity is known to be one of the major causes of erroneous dielectric property measurements. Therefore, an accurate and specified definition of sensing depth can contribute towards minimizing the error in the characterization of dielectric properties. To this end, this work investigates the sensing depth of 2.2 mm-diameter open-ended coaxial probe for skin cancer detection. While measuring in-vivo dielectric properties of the skin tissue it must be noted that the skin is composed of layers; therefore, it is heterogeneous and the sensing depth of the probe must be defined for accurate determination of potential abnormalities such as early-stage moles that can potentially be malignant and curable when diagnosed early. In this study, simulations and experiments were performed for sensing depth analysis with double-layered configuration samples. Unlike previously reported studies, the experiments consisted of a simple measurement setup without forming a special experiment setup tank. The double-layered samples included a skin mimicking phantom as a first layer and liquids with low dielectric properties; that is, olive oil and triton X-100, were added as second layers. The thickness of each layer was calculated before dielectric properties were measured in order to calculate the measurement step size and a reference level was set to eliminate probe pressure-related errors. Liquids with low dielectric properties provide substantial insight for a potential change in measured dielectric properties since the liquids mimic the effect of the keratin layer which covers the top of the skin tissue. Three percent thresholds in relative permittivity changes 5, 20, and 80% were tracked to define the sensing depth at multiple frequencies. Both in the simulation and experiment results, at 2 GHz 5%, 20% and 80% increase in dielectric properties of olive oil and triton X-100 was observed when the probe tip distances are 0.66–1.2 mm, 0.36–0.86 mm and 0.14–0.42 mm, respectively, from the first layer (first reference point; that is, the top surface of skin phantom). Based on the results, we can state that a membrane layer or keratin layer on the skin tissue will potentially affect the measurement dielectric property results by 80%. These findings also suggest that the layer that is in immediate contact with the probe tip will have the most significant effect on dielectric property measurements.

## Figures and Tables

**Table 1:** Relative permittivity and conductivity measurements of each layer in double-layered configurations at 0.5 GHz and 4 GHz.

Sample	Relative Permittivity		Conductivity (S/m)	
	0.5 GHz	4.02 GHz	0.5 GHz	4.02 GHz
Skin Phantom	38.26	32.89	0.98	2.30
Olive Oil	2.56	2.26	0.02	0.04
Triton X-100	5.86	3.85	0.04	0.21

**Table 2:** Comparison of the literature data, simulation, and experiment results for skin mimicking phantom at 2 GHz.

Property	Literature [7]	Simulation [24]	Experiment
Relative Permittivity	43.54	41.72	37.04
Conductivity (S/m)	1.33	0.37	1.49

**Table 3:** Reported sensing depth analysis of open-ended coaxial probe with 2.2 mm aperture diameter utilizing double-layered configuration.

Definition	First Layer	Second Layer	Probe Aperture	Distance	
Sensing volume [20]	Teflon block ($\epsilon_{r}$ = 2)	water	2.2 mm	50% 0.2 mm *	90% 0.5 mm *
		0.9% clinical saline			
Histology depth [21]	Rubber A ($\epsilon_{r}$ = 7 at 5 GHz)	0.9% saline	2.2 mm	1.20 to 3.65 mm **	
	Rubber B ($\epsilon_{r}$ = 52 at 5 GHz)				
	Rubber A ($\epsilon_{r}$ = 7 at 5 GHz)	Fat ($\epsilon_{r}$ = 2.5 at 5 GHz)			
	Rubber B ($\epsilon_{r}$ = 52 at 5 GHz)				
	Porcine Muscle ($\epsilon_{r}$ = 46 at 5 GHz)				
	Porcine Fat ($\epsilon_{r}$ = 11 at 5 GHz)				

* The distance where the measured relative permittivity reflects the 50% and 90% of the second layer relative permittivity. ** The distance where the measured relative permittivity fully reflects the second layer.

**Table 4:** Maximum dielectric property discrepancy between retrieved and Debye model for pure materials (skin, triton X-100 and olive oil) used for double-layered configurations in simulations.

Materials	Discrepancy for $\epsilon_{r}$	Frequency (GHz)	Discrepancy for σ (S/m)	Frequency (GHz)
Skin	1.00	6	0.31	6
Triton X-100	0.68	0.5	0.05	2
Olive oil	0.29	1	0.11	6

**Table 5:** Distances at the specified thresholds for simulated skin–triton X-100 configuration at 0.5, 2, 4, 10 and 20 GHz frequency points.

Frequency	Distance (mm) for 5% Increase	Distance (mm) for 20% Increase	Distance (mm) for 80% Increase
0.5	1.00	0.36	0.24
2	1.20	0.56	0.17
4	0.96	0.56	0.21
10	0.76	0.41	0.14
20	0.76	0.36	0.15

**Table 6:** Distances at the specified thresholds for simulated skin–olive oil configuration at 0.5, 2, 4, 10 and 20 GHz frequency points.

Frequency	Distance (mm) for 5% Increase	Distance (mm) for 20% Increase	Distance (mm) for 80% Increase
0.5	1	0.86	0.36
2	0.76	0.56	0.26
4	0.71	0.46	0.21
10	0.66	0.41	0.21
20	0.66	0.36	0.16

**Table 7:** Specified three levels for measured relative permittivity of skin–triton X-100 and skin–olive oil configurations at 0.5, 1, 2.04, 4.02 and 6 GHz frequency points.

Materials	Distance (mm) for 5% Increase	Distance (mm) for 20% Increase	Distance (mm) for 80% Increase
Skin–Triton X-100	0.87	0.58–0.66	0.36–0.42
Skin–Olive oil	0.81	0.6	0.42
